# Children’s DAT1 Polymorphism Moderates the Relationship Between Parents’ Psychological Profiles, Children’s DAT Methylation, and Their Emotional/Behavioral Functioning in a Normative Sample

**DOI:** 10.3390/ijerph16142567

**Published:** 2019-07-18

**Authors:** Silvia Cimino, Luca Cerniglia, Giulia Ballarotto, Eleonora Marzilli, Esterina Pascale, Claudio D’Addario, Walter Adriani, Angelo Giovanni Icro Maremmani, Renata Tambelli

**Affiliations:** 1 Department of Dynamic and Clinical Psychology, Sapienza University of Rome, 00186 Rome, Italy; 2 Faculty of Psychology, International Telematic University Uninettuno, 00186 Rome, Italy; 3 Department of Medical Surgical Sciences and Biotechnology, Sapienza University of Rome, 00186 Rome, Italy; 4 Faculty of Bioscience and Technology for Food, Agriculture and Environment, University of Teramo, 64100 Teramo, Italy; 5 Center for Behavioral Sciences and Mental Health, Istituto Superiore di Sanità, 00186 Rome, Italy; 6 Department of Psychiatry, North-Western Tuscany Region Local Health Unit, 55049 Viareggio, Italy

**Keywords:** methylation, dopamine transporter, genotype, psychopathological symptoms

## Abstract

Parental psychopathological risk is considered as one of the most crucial features associated with epigenetic modifications in offspring, which in turn are thought to be related to their emotional/behavioral profiles. The dopamine active transporter (DAT) gene is suggested to play a significant role in affective/behavioral regulation. On the basis of the previous literature, we aimed at verifying whether children’s DAT1 polymorphisms moderated the relationship between parents’ psychological profiles, children’s emotional/behavioral functioning, and DAT1 methylation in a normative sample of 79 families with school-age children (Ntot = 237). Children’s biological samples were collected through buccal swabs, while Symptom Check-List-90 item Revised, Adult Self Report, and Child Behavior Check-List/6–18 was administered to assess parental and children’s psychological functioning. We found that higher maternal externalizing problems predicted the following: higher levels of children’s DAT1 methylation at M1, but only among children with 10/10 genotype; higher levels of methylation at M2 among children with 10/10 genotype; while lower levels for children with a 9-repeat allele. There was also a positive relationship between fathers’ externalizing problems and children’s externalizing problems, only for children with a 9-repeat allele. Our findings support emerging evidence of the complex interplay between genetic and environmental factors in shaping children’ emotional/behavioral functioning, contributing to the knowledge of risk variables for a child’s development and psychological well-being.

## 1. Introduction

In recent decades, a growing amount of research has underlined the complex interplay between genes and the environment in shaping children’s development and emotional/behavioral adaptive functioning [1,2,3]. 

Several studies have underlined that children’s emotional/behavioral problems are predicted by children’s genetically-based features (for example, quantitative behavior-genetic research; see the work of [4]) and by exposure during childhood to potentially negative experiences (such as marital conflict, parental psychopathological risk, and poor quality of parent–infant relationships; see the works of [5,6,7]).

On the basis of these studies, recent literature has emphasized the mutual interaction between genetic and environmental factors in the development and maintenance of children’s psychological outcomes [8,9], underlining the role of differential susceptibility [10,11] in response to exposure to certain environments, in terms of both increased risk and resilience [12]. 

In this field, the developmental psychopathology theoretical framework [13] offers a valid model for conceptualizing the course of evolutionary trajectories, considering development as a result of reciprocal influences across multiple interacting levels of analysis [14] spanning from a child’s inherited genetic vulnerabilities to early experiences, particularly in family contexts [15]. 

Specifically, environmental factors can influence genetic activity in sequential events known as “developmental cascades” [16], which refers to the cumulative effects of interactions and transactions on development occurring between variables, within and across domains or levels of functioning, and across different systems or generations [17].

Recently, research in psychiatric genetics has moved to more complex models of psychopathology incorporating a focus on gene–environment and epigenetics interactions, suggesting that the greatest understanding of children’s adaptive functioning is realized considering the role played by polymorphic and/or epigenetic variations within specific genes, and their complex interplay with the environment provided by parents [2,18]. Regarding environmental variables, several studies have focused on parental psychopathology [19], while fewer studies have investigated maternal and paternal psychopathological risk [20] or their emotional/behavioral functioning in samples belonging to the general population [21].

### 1.1. Dopamine Transporter Gene

Genes influencing dopaminergic neurotransmission have been underlined as one of the most important candidate genes for children internalizing and externalizing problems [22], given the involvement of dopamine (DA) in the regulation of attention and cognition [23], mood and reward [24], and decision-making processes [25]. The availability of dopamine at the synaptic level is primarily modulated by dopamine active transporter (DAT), a solute carrier protein that actively pumps DA from the extracellular space into the presynaptic neuron [26]. 

The human DAT1 gene has a polymorphic 40-base pair (bp) variable number of tandem repeats (VNTR) in the 3′-untranslated region (3′ VNTR) of chromosome 5p15.3 of the dopamine transporter gene (SLC6A3). Generally, the 40-bp VNTR element can be repeated 3–11 times, but it has been shown that the greatest frequency polymorphisms are 9- or 10-repeat alleles [27,28].

Given that DAT1 plays an important role in affective and behavioral regulation, it is not surprising that several studies on clinical samples have reported significant associations of DAT with various developmental psychopathologies such as the attention-deficit/hyperactivity disorder (ADHD; [29,30,31]), conduct disorder [32], post-traumatic stress disorder [33], oppositional defiant disorder [34], autism spectrum disorder [35], and pediatric bipolar disorder [36].

However, to date, genetic association studies have reported conflicting findings regarding the specific polymorphism that can be considered “at risk” [29,37,38]. Consequently, the results of traditional genetic research also support the importance of considering the complex interplay between the DAT1 gene and the environment provided by parents, both in terms of genotype–environmental interactions (GxE) and epigenetic pattern [39], which are considered the key processes to explain the long-term effects of early contextual influences on gene expression and the resulting changes in child development, including an increased risk to psychopathology [40,41].

### 1.2. Gene x Environment Interactions for DAT1

Gene–environmental interaction (GxE) refers to any situation in which (a) the effect of a genetic variation (i.e., genotype) on psychopathological risk (e.g., internalizing and externalizing problems) depends on the presence of specific environmental experiences, and/or (b) the effect of the environment (e.g., poor parenting, parental conflicts, parental psychopathological risk) on emotional/behavioral functioning is conditional on an individual’s genotype [42].

With regard to DAT1 genotype x environment interactions, research has shown that children’s DAT1 polymorphisms moderated the relationship between both quality of maternal parenting [32,43,44,45] and maternal history of maltreatment [46] with children’s emotional/behavioral problems. 

Overall, although GxE studies have underlined that environmental effects on children’s development are dependent on the children’s genetic features [46], the findings are scarce and inconsistent. Recent advances in genetic research have shown that the impact of the interaction between genetic and environmental factors on children’s emotional/behavioral functioning might be mediated by epigenetic mechanism of DNA methylation, suggesting a need for more attention to these processes [47,48,49,50]. 

### 1.3. Beyond GxE: The Role of DAT1 Methylation

DNA methylation represents another type of gene–environment interplay in which environmental factors regulate the genomic functioning and lead to manifestations of various phenotypes during development [41]. Indeed, although epigenetic mechanisms are potentially heritable, they can be affected by various adverse life experiences, especially those early in life [51].

DNA methylation is considered a key process to explain the biology of gene–environmental interplay and the long-term effects of parental influences on gene expression, as well as the consequent changes in child development including an increased risk to maladaptive emotional/behavioral functioning [40,41,52]. Generally, human DNA methylation of the genome occurs on cytosines located in CpG sites [53], and when this occurs at the level of the promoter region of the genome, it can lead to the silencing of the genes involved [54]. 

The promoter region of the human DAT gene, particularly the 5′-untranslated region (5′-UTR), has been shown to have high sensitivity to epigenetic modifications [55], but nevertheless, epigenetic research on this gene is still scarce.

Although some studies have highlighted the existence of a close association between the methylation status of DAT and psychopathological difficulties in clinical populations of adolescents and adults [56,57], only few studies have investigated this relationship in school-age children [20,31,58]. For example, in a sample of children with ADHD, Xu et al. [59,60] found only an association between the methylation of CpG site 1 of the DRD4, but not with DAT1. On the other hand, a study by Ding et al. [58] reported a significant association between children’s ADHD symptoms’ response to a methylphenidate treatment (MPH) with low DNA methylation of DAT1. Our previous study [31] on a sample of children diagnosed with ADHD has shown significantly lower levels of DAT methylation in diagnosed group compared to a control group. Furthermore, this study found that children’s DAT methylation was correlated to the gravity of symptoms, but this association was conditional on the children’s polymorphisms (i.e., DAT 10/10 genotype; [31]).

To date, there is a dearth of studies focused on the role played by DAT methylation and genotype on psychological profile in a community sample of school-age children [20]. Emerging evidence in the field of developmental psychopathology has underlined the importance of improving research on the general population of school-age children, because in this developmental stage, psychopathological problems often tend to exist subthreshold, but they still lead to a maladjustment of the child [21]. In fact, recent studies have demonstrated that some areas of the brain (such as the parietal lobes and frontal lobes) undergo highly significant modifications after the first five years of life and well before adolescence (see, for example, the work of [60]). These areas have been suggested as part of the neurobiological substratum responsible for the correct functioning of the reward circuitry and affective regulation [61].

Recently, our study [21] explored the relationships between parental psychopathological risk, children’s DAT methylation, and their emotional/behavioral functioning, in a community sample of school-aged children. This study found significant associations, but it did not consider the possible moderating role of the children’s genotypes. 

### 1.4. The Present Study

As seen in the Introduction, parental psychopathological risk may have an effect on children’s emotional/behavioral functioning, with an important role played by genetic and epigenetic factors. However, most of these studies focused on animal samples [62] or clinical samples (for example, children with ADHD, conduct disorders, autism spectrum disorder) [35,58,63]. 

Given these solid results, the present study aims to verify the possible moderator role of genetic polymorphism of the DAT1 in the relationship between parents’ psychopathological risk on children’s emotional/behavioral functioning and on children DAT methylation, in a normative sample of families with school-age children. Previous studies have verified this effect considering serotonin [64], but no study to our best knowledge has done so with DAT. Furthermore, the international research reported mixed findings regarding DAT1 polymorphism associated with negative developmental outcomes [29,43,63,65]. Most previous studies in this field [31,45,66] have generally contrasted children with a 9-repeat allele to those without. Consequently, we reported results on children with (9/9, 9/10) and without (10/10) a 9-repeat allele. 

We acknowledge that a longitudinal study design represents the elective methodology to establish causal relationships between variables [67]. However, it has been underlined that the presence of a strong background knowledge (as in this case) may be used to define the hypothesized causal ordering of variables [68,69] and to assess possible cause–effect conclusion also by cross-sectional studies [70].

On the basis of the above premises and literature gap, the present study aimed to investigate if children’s genotype can moderate the following:

(1) The possible predictive effect of parental psychological profile on children’s DAT methylation;
(2) The possible predictive effect of the influence of children’s DAT methylation status on their own emotional/behavioral functioning;
(3) The possible relationship between parents’ psychological profile and children’s emotional/behavioral functioning.

## 2. Materials and Methods

### 2.1. Procedure and Sample

Thanks to the collaboration with primary schools of Central Italy, we recruited 117 families (composed of mothers, fathers, and one child) with children aged from 6 to 11 years old. From the total sample, we excluded families in which one or both parents could not understand the Italian language (*N* = 5); families in which parents were not the biological parents of the child (*N* = 3); families with children with mental and/or physical disabilities (*N* = 5); families in which one or more members were following a pharmacological or psychological treatment (*N* = 12); families who did not complete all the questionnaires (*N* = 6); and families who refused to participate in the study (*N* = 7).

The final sample was composed of 79 children (43 females and 36 males with age ranging from 6 to 11 years; M = 7.78 years; SD = 1.57), their mothers (M = 41.53 years; SD = 4.86), and their fathers (M = 43.83 years, SD = 5.10). The families were 100% Caucasian, and most of the families (88.61%) had a middle–high socioeconomic level according to the Hollingshead’s social status index [71]. A large majority (97.47%) of families were intact family groups. Furthermore, 86.07% of children were first-born for both parents.

Confounding variables (such as alcohol use, smoking, drugs of abuse, current medical illness, traumatic experiences, and social-economic status) were assessed through an anamnestic questionnaire specifically created for this study.

After receiving the consent of the primary school headmaster, a group of psychologists specifically trained for the purposes of the study presented the project to families. Written informed consent, which explained the scope and phases of the study, was obtained by parents, and children were orally informed. Furthermore, in accordance with the Declaration of Helsinki, this study was approved by the Ethical Committee of the Department of Dynamic and Clinical Psychology at Sapienza, University of Rome (protocol number 27/2016).

#### Procedure for Biological Sampling

Children were assessed with buccal swabs (Isohelix Swab Pack, Cell Product Ltd, Harriestam, UK). Buccal cell sampling is a feasible, non-invasive method that yields reproducible results in DNA methylation studies. While this method is widely used, and is particularly appropriate for community samples, it should be noted that other studies have used other methods [72]. Parents were aware that their children should not eat (including chewing gum, candy, and so on), drink (except water), and brush their teeth for at least 1 h before sampling.

Epithelial cell samples were carefully collected through the buccal swabs. The biological samplings were slightly chilled by Normative ice (+4 °C) and transported to the laboratories of the co-author (E.P.) for further processing. After buccal swabs were gathered, mothers and fathers independently filled out self-report and report form questionnaires (described below). The order of administration of these measures was randomly selected. The following tools were chosen because they are very widely used and proved to be able to capture a wide range of difficulties that can be experienced by adults and children in the general population [73,74].

### 2.2. Instruments

#### 2.2.1. Assessment of Parents’ Psychopathological Symptoms

Parents were administered the Symptom Check-List-90 item Revised (SCL-90-R), a 90-item self-report questionnaire. It measures psychological symptoms and psychological distress in adults from general and clinical populations [75]. The SCL-90-R is rated on a Likert scale of 0 (not at all) to 4 (extremely), and asks participants to report if they have suffered in the past week from symptoms of somatisation (e.g., headaches), obsessive-compulsivity (e.g., having to check and double-check what you do), interpersonal sensitivity (e.g., feeling that people are unfriendly or dislike you), depression (e.g., feeling blue), anxiety scale (e.g., feeling fearful), hostility (e.g., having urges to beat, injure, or harm someone), phobic anxiety (e.g., feeling afraid to go out of your house alone), paranoid ideation (e.g., the idea that you should be punished for your sins), and psychoticism (e.g., having thoughts that are not your own). Aside from these nine primary scales, the questionnaire provides a global severity index (GSI), which is used to determine the severity and degree of psychological distress. The SCL-90-R showed good internal coherence (α = 0.88) in this study (Italian validated version, [73]). In this study, we used the GSI scores to assess parents’ psychopathological risk.

#### 2.2.2. Assessment of Parents’ Emotional/Behavioral Functioning

Parents were administered the Adult Self Report (ASR; [76]). The ASR for ages 18–59 is a self-report, paper-and-pencil survey used to elicit information regarding psychological functioning. Items are assessed on a three-point Likert scale (0 = not true, 1 = somewhat true or sometimes true, and 2 = very often or very true). For the current study, two narrowband scales were utilized as indicators for the latent constructs of internalizing and externalizing behavior problems, respectively. Specifically, the indicators of externalizing behavior problems were as follows: Aggressive Behavior (e.g., “I argue a lot” and “I am mean to others”), Rule-Breaking Behavior (e.g., “I destroy my own things” and “I act without stopping to think”), and Intrusive Behavior (“I brag” and “I try to get a lot of attention”).

Similarly, indicators of internalizing behavior problems were as follows: Anxious/Depressed (e.g., “I feel that no one loves me” and “I cry a lot”), Withdrawn (e.g., “I am not liked by other kids” and “I keep from getting involved with others”), and Somatic Complaints (e.g., “I feel dizzy or lightheaded” and “I feel overtired without good reason”). Research has demonstrated good reliability and validity for the ASR scales (α = 0.75–8) [76]. Associations between cross-informant data have been examined for the ASR, but to our knowledge, dyadic properties have not been examined.

#### 2.2.3. Assessment of Children’s Emotional/Behavioral Functioning

Parents also filled out the Italian version of the Child Behavior CheckList/6–18 (CBCL/6-18; [77,78]), which is one of the most widely used instruments to assess child and adolescent psychopathology in both epidemiological and clinical samples. The CBCL/6–18 is a 113-item informant-report questionnaire that asks parents (mothers and fathers independently) to rate specific emotional/behavioral problems of their child during the past six months. Items are rated on a three-point Likert scale ranging from 0 (not true) to 2 (very true or often true), and they are grouped into eight empirically based syndrome scales: anxious/depressed, withdrawn/depressed, somatic complaints, social problems, thought problems, attention problems, rule-breaking behavior, and aggressive behavior. These subscales, in turn, combine into three broad-band scales: internalizing problems (comprised of items from the anxious/depressed, withdrawn-depressed, and somatic complaints scores) and externalizing problems (which combines rule-breaking and aggressive behavior). There also is a total problems score, which comprises the scores from all problem items. 

For the aims of this study, statistical analyses were performed on raw scores. As suggested by several studies [79,80,81], data were obtained by mothers and fathers (independently). The international literature has emphasized that parents can be discordant in the observation of their children. However, for this study, the mean scores of the internalizing, externalizing problems, and total problems reported by mothers and fathers were used [82]. To do this, we have primarily carried out analysis of variance (ANOVA) to verify the absence of significant differences between CBCL/6–18 scores reported by mothers and fathers (all *p* > 0.05). Mean test–retest reliabilities of r = 0.88 were reported for the school-age forms [77]. The CBCL/6-18 showed a good internal coherence coefficients and its validity has been evidenced in differentiate between clinical and general populations of children [77].

#### 2.2.4. DNA Isolation and Genotyping

Buccal cell DNA isolations were performed using the Buccal-Prep Plus DNA isolation kit (Isohelix, Cell Product Ltd., Harriestam, UK) according to the manufacturer’s instructions. The yield of DNA is usually between 3 and 10 µg. The 3′-UTR repeated sequence of DAT was amplified by the polymerase chain reaction (PCR) as described previously [29,31].

#### 2.2.5. Analysis of DNA Methylation

DNA from the buccal swabs were further processed for assessing the amount of methylation in the DAT1 5′-UTR sequence (these sites fall within a “CpG island”; notably, not in the transcription promoter region). The amount of methylation was determined in six specific CpG residues (termed M1, M2, M3, M5, M6, and M7). Notably, M1–M3 represent a CGGCGGCGG motif, while M5/M6 represent a CGCG motif. The following primers (5′–3′) were used to amplify the gene for DAT: Fwd, AGCTACCATGCCCTA TGTGG; Rev, ATCAGCACTCCAAACCCAAC. Bisulfite-treated DNA was amplified by PyroMark PCR Kit (Qiagen, Hilden, Germany) in accordance with the manufacturer’s protocol. PCR conditions were as follows: 95 °C for 15 min, followed by 45 cycles of 94 °C for 30 s, 56 °C for 30 s, 72 °C for 30 s, and 72 °C for 10 min. PCR products were verified by agarose electrophoresis.

Pyrosequencing methylation analysis was conducted using PyroMark Q24 (Qiagen, Hilden, Germany). The level of methylation was analysed using PyroMark Q24 Software (Qiagen, Hilden, Germany), which calculates the methylation percentage [mC/(mC + C)] for each CpG site, allowing quantitative comparisons (mC is methylated cytosine and C is unmethylated cytosine). In the last decade, the hydroximethylation status of CpG residues has been proposed as another important epigenetic modification induced by the environment. The pyrosequencing methylation analysis that was conducted in this study is not able to detect or distinguish hydroxi- from common methylation.

### 2.3. Statistical Analysis

After verifying the presence of correlations between parents’ and children’s psychological and epigenetic variables, hierarchical regression analyses were carried out. Specifically, on the base of our first aim, we carried out hierarchical regression to test the hypothesis that children’s DAT1 genotype (i.e., 10/10 polymorphism) moderated the predictive effect of parental psychological profile (parents’ scores at SCL-90-R and ASR; independent variables) on children’s DAT methylation (average scores of maternal and paternal CBCL/6-18; dependent variables). 

Furthermore, we carried out hierarchical regression to verify the hypothesis of the presence of a moderation effect of children’s DAT genotype (i.e., 10/10 polymorphism) on the relationship between children’s DAT methylation status (at all CpG considered; independent variables) on their own emotional-behavioral functioning (dependent variables). 

Finally, given that several studies have shown that gene–environment interactions can be confounded by the presence of gene–environment correlations (rGE; [83,84]), as suggested by Richards et al. [85], we primarily carried out Pearson correlation analyses. Then, a hierarchical regression was carried out to verify if children’s DAT1 polymorphisms moderated the relationship between parents’ psychological profile (independent variable) and children’s emotional/behavioral functioning (dependent variable).

Moderation analyses were conducted utilising the PROCESS macro for SPSS [86] and all results were adjusted to control for confounding variables including concurrent medical illness, use of medicines, and traumatic experiences. All analyses were performed with Statistical Package for the Social Sciences, SPSS software, version 25 (IBM, Chicago, IL, USA).

## 3. Results

### 3.1. The Predictive Effect of Parents’ Psychological Profile on Children’s DAT Methylation, Moderated by Children’s DAT1 Genotype

In order to test our first hypothesis that children’s DAT1 genotypes would moderate the predictive effect of parental psychological profile on children’s DAT methylation, we carried out hierarchical regression analyses for all six selected children’s DAT methylation CpG sites.

In the first step of hierarchical regression, mothers’ and fathers’ scores on the SCL-90-R GSI, ASR Internalizing and Externalizing Problems, and children’s DAT1 genotype were entered. In the second step, we included interaction terms between children’s genotype and all parental psychological variables considered in step 1.

For children’s methylation of DAT1 gene at CpG M1, the results showed no significant predictors at the first step, but in step 2, it was predicted by the father’s externalizing problems (*β* = 1.02; *t* = 3.14; *p* = 0.003) and by the interaction between children’s DAT1 genotype and maternal externalizing problems (*β* = 0.54; *t* = 2.93; *p* = 0.006). This model explained 37% of the variance (R^2^ = 0.377).

In step 1, children’s methylation at CpG M2 was predicted by the mother’s internalizing problems (*β* = 0.62; *t* = 2.90; *p* = 0.006). In step 2, there were significant negative predictions of mothers’ externalizing problems (*β* = −0.43; *t* = −2.50; *p* = 0.01) and a significant interaction between maternal externalizing problems and children’s genotype (*β* = 0.37; *t* = 2.18; *p* = 0.03). This model explained 46% of the variance. The first step accounted for 20.5% of the variance, and the second step accounted for an additional 25.5% of the variance.

We also found that for children’s methylation at CpG M3 in step 1, there was a positive predictive effect of maternal internalizing problems (*β* = −0.74; *t* = 3.50; *p* = 0.00) and a negative predictive effect of maternal global severity index (*β* = −0.43; *t* = −2.27; *p* = 0.02). In step 2, the mother’s externalizing problems was a negative predictor (*β* = −0.34; *t* = −2.10; *p* = 0.04), and there was also a significant interaction between maternal internalizing problems and children’s genotype (*β* = 0.89; *t* = 2.81; *p* = 0.00). Step 1 accounted for 23.5% of the variance, while the second step accounted for an additional 27% of the variance.

Moreover, children’s M5 methylation was negatively predicted by mothers’ internalizing problems (R^2^ = 0.285; *β* = −0.49; *t* = −2.40; *p* = 0.02) in step 1, and by fathers’ externalizing problems (R^2^ = 0.447; *β* = 0.80; *t* = 2.59; *p* = 0.01) in step 2. Interestingly, children’s genotypes were a significant predictor of methylation status at M5 CpG site in both step 1 (*β* = 0.404; *t* = 2.95; *p* = 0.005) and step 2 (*β* = 0.34; *t* = 2.48; *p* = 0.018). Hence, children with a 10/10 genotype have significantly more methylation at M5 than children with a 9-repeat allele (*p* < 0.05).

**Figure 1:**
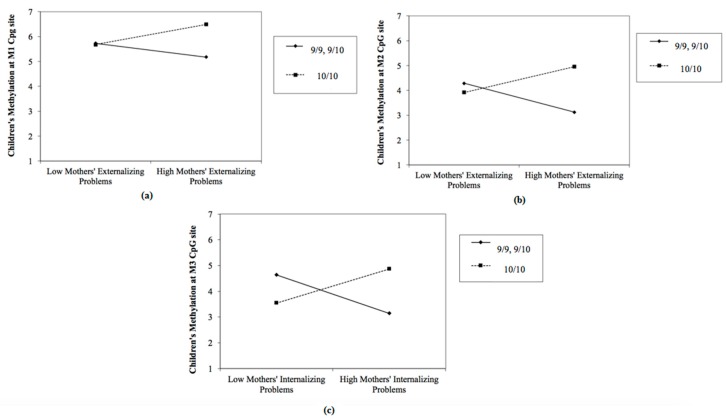
Scatter plot showing the moderation of children’s dopamine active transporter 1 (DAT1) genotype (10/10 genotype contrasted with combined 9/9 and 9/10 genotypes) on the relationship between maternal emotional/behavioral functioning and children’s DAT methylation at (**a**) M1 CpG sits; (**b**) M2 CpG site; and (**c**) M3 CpG site.

Finally, for children’s methylation at M7, we found no significant predictors in step 1. In the second step, there were negative predictions of maternal internalizing problems (*β* = −0.73; *t* = −2.15; *p* = 0.03) and of paternal internalizing problems (*β* = −0.99; *t* = −2.67; *p* = 0.01), and a positive prediction of paternal externalizing problems (*β* = 0.97; *t* = 2.86; *p* = 0.00). The model accounted for 33% of the variance. Moderation analyses were conducted with PROCESS [87]. Significant GxE interactions are reported in Figure 1.

Specifically, the results showed that maternal externalizing problems predicted children’s DAT1 methylation at M1, but only among children with a 10/10 genotype (*B* = 0.62; *SE* = 0.12; *t* = 2.24; *p* = 0.02; *9/9, 9/10 B* = −0.37; *SE* = −0.06; *t* = −0.69; *p* = 0.48) (Figure 1a). Moreover, for children with a 9-repeat allele, mothers’ externalizing problems negatively predicted children’s methylation at M2 (*B* = −0.23; *SE* = 0.10; *t* = −2.30; *p* = 0.02). Conversely, there was a positive interaction effect for children with a 10/10 genotype (*B* = 0.30; *SE* = 0.14; *t* = 2.21; *p* = 0.03) (Figure 1b). Finally, internalizing problems of mothers significantly predicted children’s methylation at M3, but only in the presence of children’s 10/10 genotype (*B* = 0.39; *SE* = 0.11; *t* = 3.44; *p* = 0.001; *9/9, 9/10 B* = −0.30; *SE* = −0.17; *t* = −1.72; *p* = 0.08) (Figure 1c).

### 3.2. The Predictive Effect of Children’s Biological Characteristics on Their Own Emotional/Behavioral Functioning, Moderated by DAT1 Genotype

In order to test our second hypotheses on the possible moderation effect of children’s DAT1 genotype on the influence of children’s DAT1 methylation status on their own emotional/behavioral functioning, we carried out hierarchical regression analyses. 

In step 1, genotype (10/10 vs. 9/10, 10/10) and DAT1 methylation status (at all considered CpG sites) were entered as independent variables, while scores from the CBCL-6/18 internalizing, externalizing, and total problems were entered as dependent variables. In step 2, the interactions between genotype and methylation status at each CpG site were entered. 

In step 1, children’s internalizing problems were predicted by children’s methylation of DAT1 at CpG M2 (*β* = 1.33; *t* = 3.25; *p* < 0.001) and M6 (*β* = 0.60; *t* = 2.16; *p* < 0.05). In step 2, the results showed a significant effect of the DAT1 genotype × M2 interaction (*β* = 1.76; *t* = 2.22; *p* < 0.05). The model explained 59.7% of the variance. Specifically, step 1 accounted for 49% of the variance, while step 2 accounted for an additional 10% of the variance. The results are shown in Table 1.

To verify the moderating effects, we used the SPSS macro PROCESS [87]. Main and interaction effects were centred to minimize multicollinearity [88].

**Figure 2:**
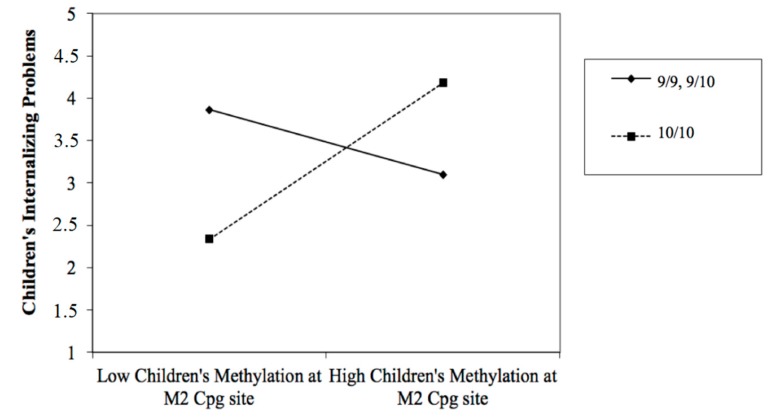
Scatter plot showing the moderation of children’s DAT1 genotype (10/10 DAT1 genotype contrasted with combined 9/9 and 9/10 genotypes) on the relationship between children’s DAT methylations (at M2, M3, and M5 CpG sites) and their internalizing problems.

Figure 2 shows the significant moderator effects of children’s DAT1 genotype. There was a significant positive relation between M2 and internalizing problems for children with 10/10 genotypes (*B* = 0.67; *SE* = 0.17; *t* = 3.86; *p* = 0.000). However, this relation was not significant for children with a 9-repeat allele (*B* = 0.02; *SE* = 0.18; *t* = 0.12; *p* = 0.89).

For children’s externalizing and total problems, no significant main or interactive effects were found (all *p* > 0.05).

### 3.3. The Predictive Effect of Parental Psychological Profile and Their Emotional/Behavioral Functioning on Children’s Emotional/Behavioral Functioning, Moderated by Children’s DAT1 Genotype

Next, our final hypothesis was that children’s DAT1 polymorphisms would moderate the relationship between parents’ psychological profile and child’s emotional/behavioral functioning.

As suggested by several studies [32,84,89,90], tests of GxE are ambiguous in the presence of gene–environment correlations (rGE), and it is necessary that gene and the environment are uncorrelated or rGE must be taken into account in the analyses. Consequently, we primarily verify for rGE, carrying out Pearson’s correlation to test the possible presence of associations between children’s genotype and parental psychological profiles. There was no significant association between children’s DAT1 genotype and parental psychological profiles (all *p* > 0.05). Consequently, hierarchical regression analyses were carried out for testing GxE. In step 1, children’s DAT1 genotype as well as mothers’ and fathers’ scores on the SCL-90-R GSI and ASR Internalizing and Externalizing Problems were entered as independent variables, while CBCL-6/18 Internalizing, Externalizing, and Total Problems scores were entered as dependent variables. In step 2, the interactions between genotype and each parental psychological variable considered in step 1 were included. 

The results showed no significant main or interactive effects for children’s internalizing problems (all *p* > 0.05). However, there was a significant predictive effect of paternal ASR externalizing problems on children’s externalizing problems (*B* = 0.51; *t* = 2.20; *p* < 0.05), and a significant interaction effect of children’s DAT1 genotype × fathers’ externalizing problems on children’s externalizing problems (*B* = −0.60; *t* = −2.07; *p* < 0.05), but only in step 2. This model explained 23% of the variance. Specifically, step 1 accounted for 14.5% of the variance, while step 2 accounted for an additional 0.08% of the variance. The results are shown in Table 2.

To verify the moderation effect, we conducted conditional process analyses using the PROCESS macro for SPSS [88], and we found a significant positive relation between fathers’ externalizing problems and children’s externalizing problems for children with a 9-repeat allele (*B* = 0.18; *SE* = 0.07; *t* = 2.44; *p* = 0.01). However, this relation was not significant for children with a 10/10 genotype (*B* = −0.02; *SE* = 0.09; *t* = −0.26; *p* = 0.78).

Furthermore, in step 2, there was a significant predictive effect of fathers’ ASR externalizing problems on children’s total problems (*B* = 0.45; *t* = 2.02; *p* < 0.05), but significant interactive effects were not found. The results are presented in Table 3.

## 4. Discussion

The present study aimed to investigate GxE interactions in families with school-age children. In particular, the study took into account the dopaminergic system, which several authors have indicated as an important system for the regulation of children’s emotional/behavioral functioning and their ability to cope with the environment [29,65]. On the basis of a bio-psycho-social model considering genetic, epigenetic, and biological measures from the psycho-environmental perspective of developmental psychopathology, the present study aimed to verify the presence of a moderator effect of children’s genotype on the relationship between parents’ psychological profiles on children’s epigenetic processes and on their emotional/behavioral functioning.

Specifically, our first hypothesis was that children’s DAT1 genotype moderated the predictive effect of parental psychological profile on children’s DAT1 methylation. To verify this moderator effect, we conducted hierarchical regression analyses for all six selected children’s DAT1 methylation CpG sites. In the first step, mothers’ and fathers’ psychological profiles and children’s DAT1 genotypes were entered. In the second step, we included interaction terms between children’s genotypes and all parental psychological variables considered in step 1. Then, significant interactive effects were examined with the PROCESS macro for SPSS.

The results showed that children’s DAT1 genotype moderated the relationship between mothers’ psychological emotional/behavioral functioning and children’s DAT methylation. In particular, higher levels of maternal externalizing problems predicted higher levels of children’s DAT1 methylation at M1, but only among children with the 10/10 genotype. Similar results were found with respect to children’s DAT methylation at M3: higher levels of maternal internalizing problems predicted higher levels of children’s DAT methylation at M3, only in children with the 10/10 genotype).

Interesting results were found on the relationship between mothers’ externalizing problems and children’s levels of DAT methylation at M2. In fact, in children with 10/10 genotypes, higher levels of mothers’ externalizing problems predicted higher levels of DAT methylation at M2, while for children with 9-repeat allele, they predicted lower levels of methylation at the same site. This finding is in line with recent literature [90], which suggested that individuals carrying specific polymorphisms would show an increase physiological reactivity of environmental risk factors on levels of methylation, mitigating or potentiating the effect of environmental exposure with respect to individual with other genotypes [91]. 

Moreover, we found also the main effects of maternal internalizing problems on children’s methylation; higher levels of mothers’ internalizing problems predicted higher levels of children’s methylation at M2 and M3, but lower levels of methylation at M5 and M7 CpG sites.

These results are consistent with recent evidence that has shown the predictive effect of environmental risk exposure in childhood (such as maternal psychopathological difficulties) on children’s DNA methylation [92]. Furthermore, in this field, molecular genetic studies [93,94] have underlined that genetic polymorphisms influence the DNA methylation, but to our knowledge, this is the first study that tested this association considering the role played by DAT1 gene.

Regarding fathers’ psychological profiles, the results showed that paternal externalizing problems positively predicted children’s DAT1 methylation at M1 and, conversely, they were a negative predictor of M7 methylation. There also was a negative main effect of paternal internalizing problems on children’s methylation at M7. Despite the growing evidence for the role played by fathers’ mental health as a risk or protective factor for child development [95,96], there is a dearth of studies specifically focused on paternal influence on children’s epigenetic processes [97]. However, our previous study [20] supports the emergent idea that the paternal psychological profile acts as an environmental risk factor for triggering children’s DNA methylation.

Finally, we found a main effect of children’s genotype on their own DAT1 methylation at M5, with a higher level of methylation on children with DAT1 10/10 genotypes. Overall, our results confirmed previous studies suggesting that environmental and genetic factors may have both a main and interactive effect on children’s methylation status [31,98]. Interestingly, our results suggested that children’s genotypes moderate children’s methylation in response to maternal (but not paternal) psychological profiles. This finding is in contrast to the idea that every environmental risk factor exacerbates genetic influences, supporting the evidence that the presence and the form of genotype moderation may vary according to specific environmental experience [99]. In our study, children’s DAT1 genotype seems to confer a greater physiological susceptibility to exposure to the maternal environment.

Our second hypothesis was that children’s DAT1 genotypes (i.e., 10/10 polymorphism) moderated the relationship between children’s DAT1 methylation status and their own emotional/behavioral functioning. To verify this moderation effect, we carried out hierarchical regression analyses. Specifically, in step 1, children’s DAT1 genotypes and methylation status were considered as independent variables, while children’s emotional/behavioral functioning was considered as a dependent variable. In step 2, the interactions between genotype and methylation status at each CpG site were considered.

The results showed that in step 1, children’s internalizing problems were predicted by children’s methylation at CpG M2 and M6. Considering the DAT1 genotype, we found positive relations between children’s methylation at CpG M2 and internalizing problems for children with 10/10 genotypes. On the other hand, this relation was not significant for children with a 9-repeat allele.

In our previous study [20], we found a positive association between the same methylation sites (M2 and M6) and children’s problems in the internalizing area. However, the present result showed that high levels of methylation in M2 predict high levels of internalizing problems, but only in children with 10/10 genotypes. This result supports recent international literature underlining that the influence of children’s epigenetic characteristics on their own emotional/behavioral functioning is conditioned by genetic features [100,101]. In particular, in a clinical sample of children diagnosed with ADHD, Adriani and colleagues [31] have shown that children’s DAT1 methylation was associated with the severity of ADHD symptoms only in the presence of a DAT1 10/10 genotype. Our results support these findings in the general population, underlining the importance of increasing focus on DAT1 polymorphisms, associated levels of DAT methylation, and their complex interplay with the environment to increase our knowledge on adaptive vulnerability and resilience during childhood and, consequently, to implement the development of more targeted and effective prevention programs.

Finally, our third hypothesis was that children’s DAT1 polymorphisms moderate the relationship between parents’ psychological profiles and children’s emotional/behavioral functioning. To verify this hypothesis, we conducted hierarchical regression analyses. In step 1, children’s DAT1 genotype and mothers’ and fathers’ psychological profiles were entered as independent variables, while children’s emotional/behavioral functioning was entered as a dependent variable. In step 2, the interactions between genotype and each parental psychological variable considered in step 1 were included.

As expected, our findings confirmed the presence of a DAT1 genotype x environment interaction on the relationship between parents’ and children’s emotional/behavioral functioning. In particular, the results showed a main effect of fathers’ externalizing problems on children’s emotional/behavioral functioning. On the other hand, the results showed a significant positive relationship between fathers’ externalizing problems and children’s externalizing problems, only in children with a 9-repeat allele (this relation was not significant in children with 10/10 polymorphism). These results are in line with Li and Lee’s findings [44]; considering the role played by the quality of parenting, their study found higher externalizing problems in children with a 9-repeat allele [44]. However, other studies have reported a higher risk associated with children’s 10/10 genotypes [32,46], underlining that the current literature in this field is unclear. On the other hand, all these studies have focused on the only role played by mothers. Our study is one of the first that took into account the environmental and genetic role played by fathers.

Nonetheless, our study did not find main or interactive effects of maternal psychological profile on children’s emotional/behavioral functioning. This result is unexpected. Indeed, a growing body of research has widely shown that maternal psychopathological difficulties predicted children’ psychopathological risk [102,103] and that children’s genotype may moderate this relationship [104,105]. However, research exploring the role played by children’s DAT1 genotype has reported mixed findings [43,44,45,63,106] and further studies should better investigate this relationship. 

This study has some limitations. In fact, we have evaluated the possible influence of parents’ psychopathological risk on children’s development, but not considering the role played by other family-related risk factors commonly associated with children’s internalizing and externalizing problems, such as couple adjustment [107,108] and parental distress [109]. Moreover, we assessed parents’ and children’s psychological profiles through report-form and self-report instruments (respectively). Although these instruments have proven their validity in several investigations and are widely used in international research, future studies should assess these variables through observational procedures and/or clinical interviews. 

Notwithstanding the above limitations, the present study has several strengths. In particular, it is one of the first to focus on school-aged children’s emotional/behavioral functioning and methylation, considering the role played by children’s genetic features (i.e., DAT1 genotype) in response to the environment provided both by mothers and fathers (i.e., parental psychological profiles). Moreover, although the previous literature suggested that specific children’s psychological problems or disorders, (e.g., ADHD, conduct disorders, autism spectrum disorder) might be more influenced by genetic and epigenetic factors than others, in this study, we chose to assess a broader range of possible maladaptive symptoms to have an in depth assessment of their psychological functioning through measures (SCL, ASR, CBCL) tapping into several problematic dimensions.

Moreover, this is one of few studies in this field that considered families of the general population [20,46,110]. This is consistent with recent international literature on developmental psychopathology that has highlighted the necessity to focus research on community samples of school-aged children. In fact, this is a life phase in which psychopathological difficulties may occur in subclinical forms [21]. 

## 5. Conclusions

The present study aimed to investigate the presence of a moderator effect of children’s DAT1 genotype on the relationship between parents’ psychological profiles on children’s epigenetic processes and on their emotional/behavioral functioning. It is one of the first to focus on school-aged children’s emotional/behavioral functioning and methylation, pondering the role played by children’s genetic features (i.e., DAT1 genotype), as well as considering parental psychological profiles. 

Our findings supported emerging evidence of the complex interplay between genetic and environmental factors in shaping children’s emotional/behavioral functioning, contributing to the knowledge of risk variables for a child’s development and psychological well-being [111]. 

Because of the cross-sectional design of this study, it is not possible to draw robust causal conclusions from our results. Therefore, future longitudinal studies are needed to support our preliminary findings, in order to contribute to the promotion of primary prevention and intervention strategies for child development.

## Figures and Tables

**Table 1:** Results of the hierarchical regression analyses predicting children’s internalizing problems. CBCL, *Child Behavior CheckList*.

Predictors	Outcome: CBCL/6-18_Internalizing Problems					
	Model 1			Model 2		
	*B*	*t*	*p*	*B*	*t*	*p*
M1	−0.35	−1.61	0.11	−0.15	−0.44	0.66
M2	1.33	3.25	0.00 **	0.05	0.07	0.94
M3	−0.59	−1.60	0.11	0.44	0.58	0.56
M5	−0.51	−1.69	0.10	−0.23	−0.45	0.65
M6	0.60	2.16	0.03 *	0.55	1.39	0.17
M7	−0.12	−0.59	0.55	−0.33	−1.09	0.28
DAT1 genotype ^a^	0.08	0.55	0.58	−0.23	−0.96	0.34
DAT1 × M1				−0.04	−0.16	0.86
DAT1 × M2				1.76	2.22	0.03 *
DAT1 × M3				−1.33	−1.75	0.09
DAT1 × M5				−0.21	−0.4	0.69
DAT1 × M6				0.68	0.94	0.35
DAT1 × M7				−0.39	−0.84	0.40
R^2^	0.495			0.597		

^a^ contrast group is 9/9, 9/10 dopamine active transporter 1 (DAT1) genotype; * *p* < 0.05; ** *p* < 0.01.

**Table 2:** Results of hierarchical regression analyses predicting children’s externalizing problems. ASR, *Adult Self Report*; SCL-90-R, *Symptom Check-List-90 item Revised*; GSI, global severity index.

Predictors	Outcome: CBCL/6-18_ Externalizing Problems					
	Model 1			Model 2		
	*B*	*t*	*p*	*B*	*t*	*p*
ASR Internalizing mothers	0.15	0.90	0.37	0.13	0.64	0.52
ASR Externalizing mothers	0.20	1.61	0.11	0.18	1,2	0.22
SCL-90-R GSI mothers	−0.02	−0.16	0.87	0.12	0.57	0.56
ASR Internalizing fathers	−0.29	−1.35	0.17	−0.47	−1.42	0.07
ASR Externalizing fathers	0.16	0.89	0.37	0.51	2.20	0.03 *
SCL-90-R GSI fathers	0.23	1.18	0.24	0.20	0.89	0.37
DAT1 genotype ^a^	−0.14	−1.22	0.22	−0.11	−0.97	0.33
DAT1 × Internalizing mothers				0.01	0.07	0.94
DAT1 × Externalizing mothers				−0.01	−0.07	0.94
DAT1 × GSI mothers				−0.16	−0.70	0.48
DAT1 × Internalizing fathers				0.30	1.32	0.19
DAT1 × Externalizing fathers				−0.60	−2.07	0.04 *
DAT1 × GSI fathers				0.09	0.44	0.66
R^2^	0.145			0.23		

^a^ contrast group is 9/9, 9/10 DAT1 genotype; * *p* < 0.05.

**Table 3:** Results of hierarchical regression analyses predicting children’s total problems.

Predictors	Outcome: CBCL/6-18_ Total Problems					
	Model 1			Model 2		
	*B*	*t*	*p*	*B*	*t*	*p*
ASR Internalizing mothers	0.01	0.09	0.92	0.04	0.22	0.82
ASR Externalizing mothers	0.11	0.94	0.34	0.20	1.34	0.18
SCL-90-R GSI mothers	0.30	1.93	0.05	0.21	1.01	0.31
ASR Internalizing fathers	−0.13	−0.64	0.52	−0.25	−1.01	0.31
ASR Externalizing fathers	0.21	1.21	0.23	−0.45	2.02	0.04 *
SCL-90-R GSI fathers	0.11	0.60	0.55	0.08	0.36	0.71
DAT1 genotype ^a^	−0.19	−1.72	0.09	−0.17	−1.53	0.13
DAT1 × Internalizing mothers				−0.05	−0.22	0.82
DAT1 × Externalizing mothers				−0.20	−1.41	0.16
DAT1 × GSI mothers				0.23	1.01	0.31
DAT1 × Internalizing fathers				0.10	0.48	0.62
DAT1 × Externalizing fathers				−0.48	−1.71	0.09
DAT1 × GSI fathers				0.15	0.73	0.46
R^2^	0.224			0.29		

^a^ contrast group is 9/9, 9/10 DAT1 genotype; * *p* < 0.05.
